# Antioxidants and Male Fertility: From Molecular Studies to Clinical Evidence

**DOI:** 10.3390/antiox8040089

**Published:** 2019-04-05

**Authors:** David Martin-Hidalgo, Maria Julia Bragado, Ana R. Batista, Pedro F. Oliveira, Marco G. Alves

**Affiliations:** 1 Unit for Multidisciplinary Research in Biomedicine (UMIB), Laboratory of Cell Biology, Department of Microscopy, Institute of Biomedical Sciences Abel Salazar (ICBAS), University of Porto, 4050-313 Porto, Portugal; pfobox@gmail.com; 2 Research Group of Intracellular Signaling and Technology of Reproduction (SINTREP), Institute of Biotechnology in Agriculture and Livestock (INBIO G+C), University of Extremadura, 10004 Cáceres, Spain; jbragado@unex.es; 3 Merck S.A., 1495-190 Algés, Portugal; ana-rita.batista@merckgroup.com; 4 i3S-Instituto de Investigação e Inovação em Saúde, University of Porto, 4200-135 Porto, Portugal; 5 Faculty of Medicine, University of Porto, 4200-319 Porto, Portugal

**Keywords:** assisted reproductive technologies, sperm ROS, pregnancy, infertility, antioxidants therapy, reproductive outcome

## Abstract

Spermatozoa are physiologically exposed to reactive oxygen species (ROS) that play a pivotal role on several sperm functions through activation of different intracellular mechanisms involved in physiological functions such as sperm capacitation associated-events. However, ROS overproduction depletes sperm antioxidant system, which leads to a condition of oxidative stress (OS). Subfertile and infertile men are known to present higher amount of ROS in the reproductive tract which causes sperm DNA damage and results in lower fertility and pregnancy rates. Thus, there is a growing number of couples seeking fertility treatment and assisted reproductive technologies (ART) due to OS-related problems in the male partner. Interestingly, although ART can be successfully used, it is also related with an increase in ROS production. This has led to a debate if antioxidants should be proposed as part of a fertility treatment in an attempt to decrease non-physiological elevated levels of ROS. However, the rationale behind oral antioxidants intake and positive effects on male reproduction outcome is only supported by few studies. In addition, it is unclear whether negative effects may arise from oral antioxidants intake. Although there are some contrasting reports, oral consumption of compounds with antioxidant activity appears to improve sperm parameters, such as motility and concentration, and decrease DNA damage, but there is not sufficient evidence that fertility rates and live birth really improve after antioxidants intake. Moreover, it depends on the type of antioxidants, treatment duration, and even the diagnostics of the man’s fertility, among other factors. Literature also suggests that the main advantage of antioxidant therapy is to extend sperm preservation to be used during ART. Herein, we discuss ROS production and its relevance in male fertility and antioxidant therapy with focus on molecular mechanisms and clinical evidence.

## 1. Introduction

The mammalian spermatozoon is a cell with a high demand for energy to perform its function. Spermatozoa obtain their energy by two main metabolic pathways: glycolysis that occurs in the principal piece of the flagellum and oxidative phosphorylation (OXPHOS) that takes place on mitochondria located at the midpiece of the flagellum [1]. Spermatozoa contain between 50 and 75 mitochondria [2] and as with any other kind of cell that performs aerobic metabolism, is associated with the production of free radicals named reactive oxygen species (ROS) that include the hydroxyl radicals (•OH), superoxide anion (•O_2_^−^), hydrogen peroxide (H_2_O_2_), and nitric oxide (NO). These ROS are highly reactive molecules due to the presence of an unpaired electron in their outer shell. In addition, they have a very short half-life in the range of nanoseconds to milliseconds. ROS are produced as a consequence of natural cell machinery and participate in the normal function of a cell. However, when ROS production overcomes cellular antioxidant defenses surpassing a physiological range, they cause deleterious effects due to oxidative stress (OS) that results in oxidation of lipids, proteins, carbohydrates, and nucleotides [3].

Male subfertility and infertility have been associated with OS. Moreover, since infertile men have lower seminal plasma antioxidant capacity in comparison with fertile men, when higher levels of ROS occur, they led to an increase of lipid peroxidation (LPO) [4]. It is well described that when ROS overproduction occurs, it induces sperm DNA damage, although they have the potential to fertilize embryo development and fertility might be disturbed [5,6]. It is unclear how this is related with the fact that nowadays infertility is becoming a worldwide health problem, where one out of six couples are under fertility treatment and thus the use of assisted reproductive technologies (ART) to overcome this problem is growing exponentially. Nevertheless, ART is not harmless and is also associated with an increase of ROS production [7]. Although there is literature focused on the effects of consumption of oral substances with antioxidant properties on sperm parameters, the purpose of this review is to discuss the efficiency of antioxidant intake as a dietary supplement as well as an additive through ART procedures to counteract excessive ROS production that leads to infertility. We will also focus on the molecular mechanisms of action of those compounds with antioxidant activity in the male reproductive system, mainly reviewing literature that relates antioxidant treatment with ART, clinical pregnancy, and live birth as final outcomes.

## 2. Sources of ROS in Spermatozoa

**Figure 1:**
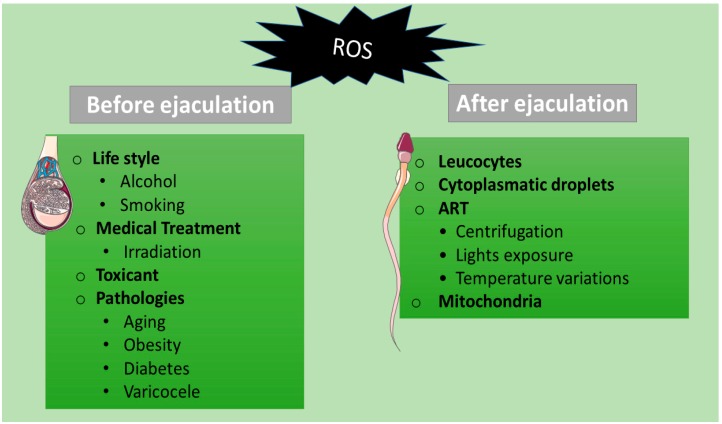
Potential stimuli that cause reactive oxygen species (ROS) production in spermatozoa.

Several situations result in nonphysiological levels of ROS overwhelming the natural scavenger systems (Figure 1). For example, lifestyle habits, such as alcohol consumption, smoking, exposure to toxicants, or pathologies such as obesity, varicocele, stress, and ageing have been associated with increased production of ROS in seminal plasma [8]. Presence of leucocytes in semen, as well as high percentage of spermatozoa with morphological anomalies [9] or immature spermatozoa with cytoplasmatic droplets containing high amount of enzymes are some examples associated to high ROS levels [9,10,11,12].

Currently, human infertility is a global health problem that has led to an exponential grown in the use of ART in the last years to overcome fertility problems. However, ART protocols imply sample centrifugation, light exposure, change of oxygen concentration, pH, or temperature, and the use of culture media with metals content that can produce hydroxyl radicals by Haber–Weiss and Fenton reactions (see explanation below). Hence, optimization of ART protocols has been proposed to minimize artificial ROS production, for instance, by decreasing *g*-force during sperm selection [13], decreasing spermatozoa incubation time during in vitro fertilization (IVF), which in turn decreases the time where aberrant spermatozoa that produce more ROS are in contact with the oocyte, and by decreasing sperm concentration or atmospheric oxygen concentration during embryo culture under in vitro conditions [7]. In order to reduce human leucocyte contamination on raw semen, paramagnetic bead technology (Dynabead^®^) can be used. Thus, magnetic beads coated with leukocyte antigen CD45 decrease leukocyte contamination [14,15], doubling the percentage of spermatozoa–oocyte penetration, as shown in a heterologous assay using hamster oocytes [16].

## 3. Bivalent Role of ROS on Sperm Function

**Figure 2:**
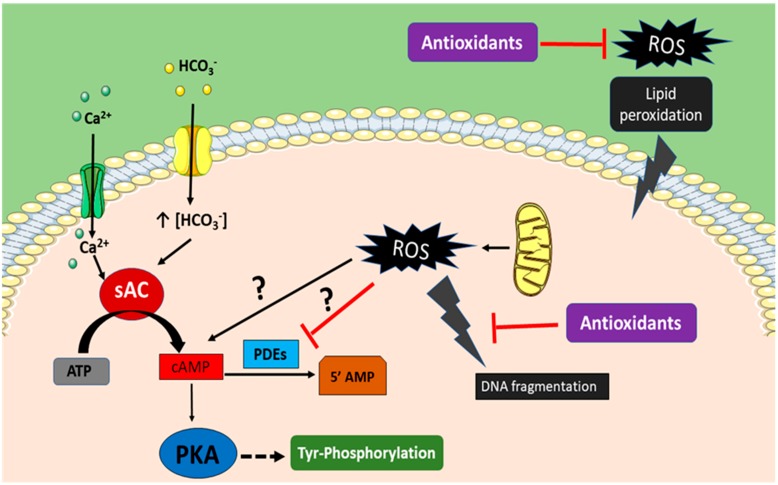
Proposed model of the bivalent role of reactive oxygen species (ROS) in sperm. (i) High levels of ROS concentration induced by different factors such as assisted reproductive technologies (ART), diseases, medical treatment, life style, etc., overwhelming the sperm antioxidant system induce plasma membrane lipid peroxidation and DNA damage. (ii) Physiological ROS level produced mainly by mitochondria induce production of high levels of cAMP by an undetermined mechanism, activating the PKA pathway, and leading to tyrosine phosphorylation, a hallmark of sperm capacitation.

Mammalian spermatozoa are extraordinary cells able to survive in a different body from where they were created. They are very specialized cells having the sole purpose to deliver the paternal genome into the oocyte. However, after ejaculation, spermatozoa must undergo a complex process within the female reproductive tract named capacitation, which allows spermatozoa to fertilize the oocyte [17,18]. Capacitation is a cascade of different cellular events that imply high production and consumption of energy. Although there is controversy on the preponderant metabolic pathways, glycolysis or OXPHOS, used by spermatozoa to generate energy in the form of ATP, it seems that there are sperm species preferences [1]. OXPHOS is the most efficient pathway, obtaining about 30 molecules of ATP by oxidizing one molecule of glucose, while during glycolysis, only two molecules of ATP are obtained per molecule of glucose. It has been described that OXPHOS is the major source of ROS in spermatozoa [19]. Furthermore, ROS might play a bivalent role in sperm function: mild ROS levels boost different intracellular events that culminate on oocyte fertilization, while higher ROS levels induce sperm DNA damage and embryo miscarriage [20,21]. In a comprehensive review, Ford summarized ROS physiological functions on sperm capacitation [22]. It is known that soluble adenylyl cyclase (sAC) is activated by bicarbonate and Ca^2+^, converting ATP into cAMP, subsequently activating the PKA pathway that mediates the phosphorylation of protein in tyrosine residues, which is used as a hallmark of sperm capacitation [23,24]. It has been proposed that ROS participate in the activation of the cAMP/PKA pathway by increasing cAMP levels, although the mechanism of cAMP production is still not clear in spermatozoa [22]. In adipocytes, it has been proposed that the mechanism of action is through inhibition of phosphodiesterase activity [25]. In human spermatozoa, it was proven that ROS action is mediated by PKA [26]. Thus, the induction of tyrosine phosphorylation was suppressed by a PKA inhibitor (H89) and the responsiveness to progesterone (sperm-oocyte fusion) when spermatozoa were coincubated with NADPH proved it to be a ROS generator [26]. In a different study, capacitated human spermatozoa showed increased levels of cAMP that was mimicked in vitro by exposure of spermatozoa to superoxide anions (O_2_^−^). Superoxide dismutase (SOD) addition inhibited cAMP levels and the sperm acrosome reaction in a concentration-dependent manner [27]. These results were confirmed by others where superoxide anions increased cAMP concentration and capacitated spermatozoa produced H_2_O_2_, leading to an increase in protein tyrosine phosphorylation [28]. Nevertheless, when ROS production overcomes antioxidant defenses, detrimental effects on spermatozoa can be summarized as increased LPO and DNA damage and reduction of sperm motility, which are associated with lower sperm fertility (Reviewed by [29]). Thus, ROS homeostasis is pivotal for male reproductive potential as they mediate important functions of sperm, such as capacitation, but when ROS levels surpass these biological levels, they readily oxidize lipids and proteins at membranes and compromise sperm quality and fertilization capacity (Figure 2).

## 4. Mechanism of ROS Defense in Spermatozoa

Spermatozoa differentiation is achieved during spermiogenesis as they gradually lose their cytoplasm. By the end of the process, the cytoplasm content is very small compared to other cells, where most of the space is occupied by DNA (sperm head). This special feature results in spermatozoa possessing low intracellular antioxidant activity consisting of superoxide dismutase (SOD), nuclear glutathione peroxidase (GPx), peroxiredoxin (PRDX), thioredoxin (TRX), and thioredoxin reductase (TRD) [30]. Therefore, sperm ROS scavenger activity basically depends on the antioxidant content of the seminal plasma, which is formed mainly by a trio of enzymes where SOD converts superoxide anion (O_2_^−^.) to hydrogen peroxide (H_2_O_2_), preventing the formation of hydroxyl radical that is an inductor of LPO. However, the H_2_O_2_ generated is a strong membrane oxidant that is rapidly eliminated either by catalase (CAT) or GPx activities, giving H_2_O as a product. Finally, seminal plasma also contains nonenzymatic antioxidant components such as α-tocopherol (vitamin E), ascorbic acid (vitamin C), pyruvate, urate, taurine, and hypotaurine [31].

It should be noted that most ART involves washing steps, meaning that all the natural antioxidant defenses contained in seminal plasma are removed. Likewise, this also happens after natural insemination. During ejaculation, spermatozoa are surrounded by antioxidant molecules coming from seminal plasma but once the ejaculate reaches the vagina, seminal plasma is diluted, leading in both cases to spermatozoa facing ROS. Although spermatozoa possess antioxidant scavenger systems, it seems that they are not strong enough when ROS levels exceed physiological levels, subsequently making spermatozoa highly susceptible to OS.

## 5. Lipid Peroxidation

**Figure 3:**
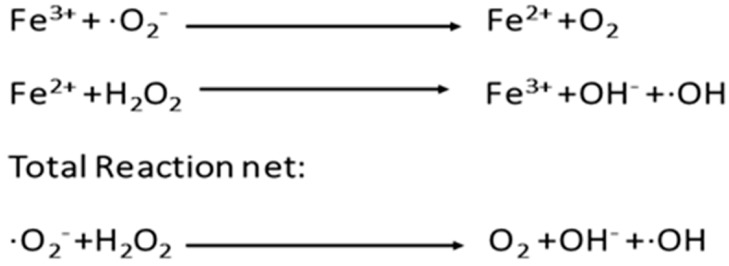
Haber–Weiss Reaction and Fenton reaction.

The sperm plasma membrane contains a high proportion of polyunsaturated fatty acid (PUFAs) to generate the fluidity needed in order to accomplish the membrane fusion events associated with fertilization. This high PUFAs content makes spermatozoa especially susceptible to suffer LPO [32,33]. The highly reactive hydroxyl radical (OH^−^) is an inductor of LPO produced through two consecutive reactions (Figure 3): the first is the Haber–Weiss reaction in which a ferric ion (Fe^3+^) in the presence of a superoxide radical (O_2_^−^) is reduced to ferrous ion (Fe^2+^), followed by Fenton reaction, where Fe^2+^ reacts with hydrogen peroxide (H_2_O_2_), forming Fe^3+^ and a hydroxyl radical (OH^−^).

Secondary products are formed during LPO: malondialdehyde (MDA), propanol, hexanol, and 4-hydroxynonenal (4-HNE) [34], which are highly reactive and may attack other nearby PUFAs, thus initiating a chain reaction with harmful effects that eventually disrupts membrane fluidity. These secondary products are used as lipid oxidative stress biomarkers.

Nowadays, cryopreservation is becoming an important issue for the success of ART in humans and livestock. Although cryopreservation is routinely used, it is a tough procedure associated with deleterious effects on sperm function due to an increase of ROS production linked to LPO and thus an increase of membrane permeability [35,36,37]. In this context, the use of antioxidants as additives during cryopreservation/thawing procedure is a common strategy to counteract negative effects of ROS on sperm function.

## 6. Effects of Oral Antioxidant Intake on Male Reproductive Outcome

Currently, there is a growing trend of oral antioxidant intake to counteract high levels of ROS found in spermatozoa and seminal plasma of subfertile or infertile men. This hypothesis is supported by several works that describe an improvement of sperm parameters after oral antioxidant intake. Among those improvements, sperm concentration, motility, or decrease of DNA damaged are reported (Reviewed by [38]). However, only a few works have shown the effect of antioxidant therapy on fertility outcomes. Here, we discuss the major findings of oral antioxidant intake in reproduction outcome and its endpoints, such as fertility and live birth (summarized in Table 1).

### 6.1. Carnitines

Carnitines are synthetized by the organism and found in seminal plasma at higher concentration than in spermatozoa. The l-carnitine (LC) isomer is the bioactive form [54] with a pivotal role in mitochondrial β-oxidation, acting as a shuttle of the activated long-chain fatty acids into the mitochondria [55] where l-acetyl-carnitine (LAC) is an acyl derivative of LC. Long-chain fatty acids provide energy to mature spermatozoa (with positive effects on sperm motility) and during maturation and the spermatogenic process [56]. Oral intake of LC (1 g twice/day) and LAC (0.5 g twice/day) for three months reduced ROS levels in spermatozoa and improved pregnancy (11.7%) in patients with abacterial prostate-vesiculo-epididymitis (PVE) with normal values of leucocytes, but it did not improve pregnancy at all (0%) in those PVE patients with high levels of leucocytes [40]. A year later, the same group tested patients diagnosed with abacterial PVE concomitant with high levels of leucocytes and showed that pretreatment for two months with a nonsteroidal anti-inflammatory followed by two months of carnitine oral intake achieved 23.1% pregnancy in comparison with the four-month carnitine intake group (0%), nonsteroidal anti-inflammatory group (6.2%), and the group receiving four-month nonsteroidal anti-inflammatory compounds and carnitines (3.8%) [41]. In another study, the effect of daily intake of LC (3 g), LAC (3 g), or a combination of LC (2 g) and LAC (1 g) was discriminated over six months and results were followed up 9 months after intervention in idiopathic asthenozoospermic men (*n* = 60) [42]. Treated men improved their total oxyradicals scavenging capacity of seminal fluid [42]. Overall, LAC or the combination of LAC + LC treatment had better improvement of sperm motility and concentration. Nevertheless, those patients with lower basal values of sperm motility had higher probability to respond to the treatment but pregnancy rate was not improved by any treatment in comparison with placebo control group [42]. Recently, coadministration of LC fumarate (2 g), LAC (1 g), and clomiphene citrate (50 mg) concurrently with vitamins and minerals in patients with idiopathic oligo- and/or asteno- and/or teratozoospermia (*n* = 173) enhanced sperm concentration specially in those patients with multiple impairment semen parameters (oligoasthenoteratozoospermic patients), but did not improve the morphology, progressive sperm motility neither pregnancy rates in comparison with control group [44]. A meta-analysis concerning carnitine used as an oral antioxidant therapy concluded that this molecule might be effective for improving pregnancy rates regarding the limits of patient inclusion criteria and the lower number of men evaluated in each study [57].

### 6.2. Vitamins

The interest of vitamin E and its use as antioxidant is due to its protective activity against ROS which subsequently decreases LPO, and therefore exerts positive effects on sperm functions, such as sperm concentration and motility [58]. However, its effects in fertility are less clear. For example, in a small clinical trial (*n* = 30), oral administration of vitamin E (300 mg twice daily) for three months raised the levels of vitamin E in blood serum, although human seminal plasma levels were not modified, questioning its possible effects on reproductive parameters [50]. Nevertheless, in this clinical trial, vitamin E treatment achieved an improvement of the zona pellucida binding test without any other improvement described, including ROS level [50]. Similarly, 15 normospermic infertile men after one month of daily consumption of 200 mg of vitamin E improved their fertilization rate (19.3 ± 23.3 pretreatment versus 29.1 ± 22.2 post-treatment) after IVF. Those results were associated with lower sperm LPO levels in comparison with preintervention values [52]. In another work, oral administration of vitamin E (100 mg thrice daily) to patients with asthenospermia (*n* = 52) established three different groups of men according to the results: (i) men without improvement of their sperm motility (40%); (ii) men with improved sperm motility but did not achieve pregnancy (39%); (iii) men with improved motility and achieved pregnancy (21%), of which 81.8% of pregnancies finished in live birth. The placebo control group did not achieve any pregnancies [47]. Later, daily intake of a combination of vitamin E and C (1 mg of each component) for two months in patients where intracytoplasmic sperm injection (ICSI) had previously failed was studied (*n* = 38). The results showed two different populations: (i) those where the antioxidant treatment decreased the percentage of sperm DNA damage (*n* = 29) and (ii) those where the treatment did not affect this parameter (*n* = 9) [49]. The most interesting result was observed in the responsive group that after ICSI, the pregnancy rate (6.9 vs. 49.3%) and implantation rate (2.2 vs. 19.2%) were improved compared with the pretreatment group, although no differences were found in embryo quality [49]. In a nonplacebo-controlled and nondouble-blind design trial, daily intake of a combination of selenium (200 µg) and vitamin E (400 UI) followed for 3.5 months by infertile men (*n* = 690) achieved 10.8% spontaneous pregnancy [50].

Several studies have been performed looking for beneficial effects from a combination compounds with antioxidant activity. For example, a formulation using a mix of several compounds with antioxidant activity (vitamin C, vitamin E, carnitine, folic acid, lycopene, selenium, and zinc) was evaluated using a mouse Gpx5 knock-out (KO) subjected to a second stress: scrotal heat (KO + SH) (42 °C for 30 min) [58]. Although the exact ingestion quantity of this antioxidant combination could not be determined, their effects include the reversion of sperm DNA oxidation induced in KO + SH animals and protection of seminiferous tubules. The results showed that animals supplemented with KO + SH versus the nonsupplemented animals had double the fertilization rate (73.7 vs. 35.2%) and fetus reabsorption was halved (8.9 vs. 17.8%) [58]. In another trial, infertile human patients with oligo- and/ or astheno- and/or teratozoospermia with or without varicocele (*n* = 104) using a combination of antioxidants (vitamin C 90 mg, vitamin B12 1.5 μg, LC 1mg, fumarate 725 mg, LAC 500 mg, fructose 1000 mg, CoQ_10_ 20 mg, zinc 10 mg, and folic acid 200 μg) were studied for six months. The results showed that the individuals from the treated group, regardless of whether they suffered from varicocele or not, presented improved sperm concentration total sperm motility [43]. Moreover, after treatment, 22.2% (10/45) of supplemented patients achieved pregnancy, while in the control group, only 4.1% (2/49) of the couples were pregnant [43]. A close analysis of the men from the supplemented group revealed that only 4.8% (1/21) of patients suffering varicocele improved after treatment, while the nonvaricocele group achieved 37.5% (9/24) pregnancy [43]. A different group studied the effect of a commercial multiantioxidant supplement (vitamin E 400 IU, vitamin C 100 mg, lycopene 6 mg, zinc 25 mg, selenium 26 μg, folate 0.5 mg, garlic 1000 mg) for three months on 60 men with high levels of DNA fragmentation and poor sperm motility and membrane integrity [51]. The treatment achieved doubled pregnancy rate (63.9 vs. 37.5%), implantation rate (46.2 vs. 24%), and viable pregnancy rate (38.5 vs. 16%) versus the placebo group without any modification of any sperm parameters, fertilization, or embryo quality rates [51]. However, this work was later criticized because of the experimental design, particularly the low number of individuals in the trial, unequal distribution of individuals between the placebo (*n* = 16) and treatment groups (*n* = 36) and the suitability of the statistical analysis used [59].

Contradictory results were found when men were supplemented with different oral antioxidants after varicocelectomy. Oral intake of vitamin E (300 mg twice/day) for 12 months (*n* = 40) improved the sperm parameters of sperm concentration and the percentage of motile spermatozoa, although these data were not significant compared with control [60]. Recently, a multiple antioxidant combo was tested (l-carnitine fumarate 1 g, acetyl-l-carnitine HCl 0.5 g, fructose 1 g, citric acid 50 mg, vitamin C 90 mg, zinc 10 mg, folic acid 200 μg, selenium 50 μg, coenzyme Q-10 20 mg, and vitamin B12 1.5 μg) after varicocelectomy (*n* = 90) for six months [45]. Surgery improved the following sperm parameters: sperm concentration, percentage of motile spermatozoa or progressive motility, and spermatozoa with normal morphology. Moreover, treated men achieved 29% pregnancy versus 17.9% in the placebo group [45].

### 6.3. Zinc

Zinc is a metalloprotein cofactor for DNA transcription and protein synthesis. Moreover, zinc is necessary for the maintenance of spermatogenesis and optimal function of the testis, prostate, and epididymis [61], in addition to their antioxidant properties preventing LPO [62]. A trial using zinc sulphate as an antioxidant therapy administrated orally (250 mg twice daily) for three months reported an improvement in the reproductive outcome of asthenozoospermic men (*n* = 100), particularly in the sperm parameters of concentration, motility, and sperm membrane integrity (hypoosmotic swelling test). It was also noticed a decrease of antisperm antibodies on seminal plasma without modification of zinc levels on seminal plasma [53]. Pregnancies were also improved in couples where men underwent treatment when compared with placebo, 22.5% (11/49) versus 4.3% (2/48), respectively [53]. In another trial with only 14 patients and no control group, sperm parameters were improved after zinc treatment (220 mg daily for four months) and 21.4% (3/14) of patients achieved pregnancy and increase zinc levels on seminal plasma [52]. Although beneficial evidence has been found on reproductive outcome after zinc intake, the lower number of studies and subjects under treatment without a proper control does not allow further discussion of the possible positive effects of zinc intake on reproduction outcome.

### 6.4. Natural Compounds—Traditional Medicine

Natural compounds have been used traditionally to treat diseases. For instance, beneficial effects on reproductive outcome have been reported using products derived from tea (*Camelia sinensis* (L.)), which is the second most consumed beverage after water [63]. For example, an in vitro experiment using green tea extract or epigallocatechin-3-gallate (EGCG) added to human spermatozoa media improved sperm capacitation hallmarks, such as tyrosine phosphorylation and cholesterol efflux, through the estrogen receptor pathway [64]. EGCG has been shown to have beneficial effects when extreme stresses are applied to male mice [65,66]. Interestingly, adverse effects induced by artificial testicular hyperthermia were ameliorated by oral administration of green tea extract [65]. Positive effects were visible after 28 days of heat stress induction, improving sperm concentration, percentage of motile and progressive spermatozoa, and sperm membrane integrity [65]. Another example of the beneficial effects of EGCG were described when intraperitoneal administration (50 mg/kg) protected against testicular injury induced by ionizing radiation in rats [66]. Thus, treated animals restored testicular function with an improvement in the number of pups by littler reducing LPO (TBARs) and protein carbonyl levels [66]. EGCG’s mechanism of action is via the mitogen-activated protein kinase/BCL2 family/caspase 3 pathway [66]. In another work, the combination of two different tea extracts, white and green, where evaluated as additives to improve ART sperm of rats stored at room temperature. The authors found doubled levels of epigallocatechin (EGC) and EGCG in white tea in comparison with green tea [67], highlighting the variability associated with the type of tea extract used. Moreover, although both extracts had positive effects, the white tea extract had better ferric reducing antioxidant power than the green tea extract and the control. The beneficial effects were proportional to the concentration used, with 1 mg/mL of white tea extract being the best concentration tested for improving sperm survival and decreasing LPO over 72 hours of storage at room temperature [67]. Encouraged by the antioxidant effects on sperm parameters of white tea, the same group explored the oral administration potential of the extract to improve prediabetic type II (PreDM) male reproduction features known to be decreased due to oxidative stress [68]. PreDM is characterized by mild hyperglycemia, glucose intolerance, and insulin resistance and has been related with infertility or subfertility problems in males [69]. Consequently, using rat as an animal model, drinking white tea counteracted the negative effects of PreDM on the male reproductive tract. For example, white tea consumption improved testicular antioxidant power and decreased lipid peroxidation and protein oxidation [68]. Ingestion of white tea also restored sperm motility and restored sperm showing morpho-anomalies to normal levels [68].

## 7. Antioxidants as a Tool to Improve Male ART Outcomes

Human infertility already affects one of six couples worldwide [70] and male factors contribute to 20–50% of infertility [71]. Infertile men tend to have higher ROS levels than fertile men. To counteract fertility problems, different ART have been developed, mainly IVF and ICSI. In both cases, gametes are extracted from the body and incubated in in vitro conditions and, after a while, an embryo is transferred into the uterus. It should be noted that due to legislation and ethical issues, it is easier to perform experiments in animal models than in humans to test antioxidant effects on different ART. The interest in the use of antioxidants to improve sperm parameters is not new. As early as 1943, in a study focused on sperm metabolism and oxygen consumption, MacLeod showed that sperm produce hydrogen peroxide, which has a deleterious effect on sperm motility, and it can be counterbalanced by addition of catalase to the media [72]. Later, some authors followed the same rationale and tried to adapt MacLeod’s hypothesis to different ART, such as cryopreservation, IVF, and ICSI.

Sperm conservation for long periods of time in liquid nitrogen (cryopreservation) is designed to keep sperm viable. From a practical point of view, cryopreservation is a tool to enable male fertility before, for example, chemotherapy, radiotherapy, vasectomy, or exposure to toxicants, or just to have time to screen donors for infectious agents, such as the human immunodeficiency or hepatitis B viruses [73]. On the other hand, from the animal industry point of view, the use of cryopreservation aims to maximize the number of services (inseminations) that can be performed from a simple ejaculation, ensuring the quality of genetical material preserved, or allowing the transportation of this genetical material to distant places. Cryopreservation is also of special interest to preserve endangered species. However, cryopreservation is not a harmless technique, inducing DNA and LPO damage and other adverse effects [74]. Moreover, cryopreservation, like ART, involves centrifugation, which is associated with production of ROS [13] and removal of seminal plasma which contains the main sperm antioxidant scavenger systems.

Antioxidant supplementation to cryopreservation media has been proposed as a way to overcome ROS production and OS status in spermatozoa (summarized in Table 2). For example, supplementation with a synthetic phenolic antioxidant, butylated hydroxytoluene (BHT), during boar sperm cryopreservation improved post-thawing sperm survival, decreased MDA levels at the concentration of 0.4 mM BHT, and embryo development was improved (28.8% vs. 15.8%) without modification of embryo cleave percentage in comparison to the control [75]. Later, it was described that 1 mM BHT improved antioxidant sperm activity, pregnancy rate (86.7 vs. 63.6%), the number of gilts farrowing (86.7 vs. 45.4%), and the number of piglets born (10.8 ± 1.6 vs. 8.2 ± 2.2) after performing intrauterine artificial insemination (IUI) using cryopreserved sperm versus control [76]. Subsequently, in a multitest in which four different compounds with antioxidant activity (BHT 2 mM, ascorbic acid 8.5 mg/mL, hypotaurine 10 mM, and cysteine 5 mM) were added during goat sperm cryopreservation, LPO was decreased but only ascorbic acid and BHT significantly improved fertility in comparison with control after performing artificial insemination (AI) [77].

The importance and the use of the amino acid cysteine in the fight against ROS impacts on the cell is due to the fact it is a limiting substrate for glutathione synthesis [90]. Cysteine (2 mM) and taurine (2 mM) (a cysteine derived) antioxidant properties were controversial when they were used during the cryopreservation procedure of bull spermatozoa [80]. Taurine decreased GSH and SOD levels, while CAT levels were five times higher than control, but MDA levels were also higher. However, cysteine increased SOD and CAT levels without an effect on MDA levels [80]. The nonreturn rate was not modified when IUI were performed by neither of the compounds; however, a nonsignificant (*p* ˃ 0.05) tendency of improvement was observed in cysteine-treated straws 74.54% (41/55) in comparison to control 57.14% (28/49) [80]. Similar results were obtained when a higher concentration of cysteine (5 mM) and trehalose (25 mM) were added again to bull cryopreservation media. Thus, the antioxidant features of these compounds were not proved; neither MDA nor GPx levels were enhanced [82]. Furthermore, no improvement on the nonreturn rate was found after IUI [82]. Similarly, using cysteamine (5 µM), a decarboxylated derivative of cysteine and lycopene (500 µg/mL) during bull sperm cryopreservation, no differences were found in the nonreturn rate [83]. In other study, the authors used N-acetyl-l-cysteine (NAC), an acetylated cysteine residue which has been shown to effectively reduce ROS formation when H_2_O_2_ stress were used in thawed bull spermatozoa [87]. However, neither sperm DNA, nor the number of blastocysts were not improved after performing ICSI using spermatozoa cryopreserved in the presence of NAC [87]. Nevertheless, in an IVF study on mice using fresh spermatozoa, where gametes and embryos were stressed by incubation under 20% oxygen atmosphere (over physiological levels on oviduct and uterine from 2–8% [91]), a combination of substances with antioxidant activity were tested (LAC 10 μM, NAC 10 μM, α-Lipoic Acid 5 μM) in either IVF media, embryo culture media, or both. Treated samples had lower intracellular levels of H_2_O_2_, accelerated embryo development, and significantly increased trophectoderm (TE) cell numbers, inner cell mass (ICM), and total cell numbers [88]. All these effects were exacerbated when the antioxidant combo were added during the whole process [88].

Positive effects were also described when thawed bull spermatozoa were supplemented with an antioxidant combination (zinc chloride 10 μg/mL, D-aspartic acid 500 μg/mL, and coenzyme-Q10 40 μg/mL), obtaining a better percentage of total sperm motile and progressive motility and a decrease of DNA fragmentation through sperm incubation [89]. Moreover, antioxidant supplementation improved embryo development. Although no differences were found in the cleave percentage, the number of blastocysts that reached the eight-cell stage was 37.1% in the control versus 51.7% in the treated group [89].

Following the rationale of MacLeod [72], adding antioxidant enzymes to counteract the adverse effects of ROS on spermatozoa was used to improve sperm cryopreservation. Enzymes with antioxidant properties were added to bull cryopreservation media—0.5 and 1.0 mM of reduced glutathione (GSH) or a combination of 0.5 mM of GSH and 100 U/mL of SOD— but did not modify the nonreturn rates [84]. In another study, the use of CAT (200 IU/mL) was used to cryopreserve ram (*Capra pyrenaica*) epidydimal spermatozoa obtained postmortem [79]. At this concentration, no differences were found in sperm parameters but negative effects were described on fertility: fewer pronucleus zygotes (25.5% control vs. 13.2% treated) and cleaved embryos were obtained from treated samples after IVF (16.7% control vs. 7.6% treated) [79].

Natural compounds with antioxidant activity have also been tested in ART. Metformin, a biguanide isolated from *Galea officialis* used worldwide as a treatment for diabetes type II [92], was recently added to the cryopreservation sperm media of chicken due to its antioxidant properties, among other properties [93]. Cryopreserved mouse spermatozoa treated with metformin displayed better motility, sperm viability, doubled fertilization rate and embryo development, and halved DNA fragmentation rate [86]. These promising results of supplementation of cryopreservation media with metformin appeared to be related to the activation of 5’AMP-activated protein kinase (AMPK). However, recently, negative results have been described when metformin (1 and 10 mM) was used to improve boar sperm preservation at 17 °C, decreasing sperm motility and mitochondria potential [94]. In an in vitro study performed in human spermatozoa kept at physiological temperature, metformin (10 mM) induced a reduction of sperm motility, where the mechanism of action was associated with PKA pathway inhibition [95]. Boar spermatozoa were coincubated during cryopreservation with rosemary extract (*Rosmarinus officinalis*) or cysteine (10 mM) or a combination of both [81]. Although both compounds enhance some sperm properties, the most noticeable effects were found by rosemary compound, enhancing total sperm motility, progressive motility, and preventing acrosome membrane damage three hours post-thawing in comparison to control [81]. Rosemary-treated spermatozoa yielded better cleave percentages without affecting blastocyst formation rate after performing IVF [81].

Melatonin (MLT) is a hormone endogenously synthesized mainly by the pineal gland. It has been detected in human seminal fluid [96] and melatonin receptors have been described in sperm of several species [97]. MLT’s antioxidant property was tested in cryopreserved human spermatozoa [98]. MLT increased the expression of the antioxidant-related gene Nrf2 as well as its downstream genes SOD2, CAT, HO-1, and GSTM1, leading to lower ROS levels and LPO [98]. On the other hand, MLT (1 µM) used during boar semen preservation at 17 °C only showed a modest membrane protective effect [99]. By contrast, cryopreserved ram sperm supplemented with MLT achieved higher viability rates, higher percentages of total motile and progressive motile spermatozoa, and higher DNA integrity [85]. However, after IVF, only faster first embryonic division without any other embryo output difference was observed in those samples supplemented with MLT [85].

Yamaguchi, et al. [100] showed that thawed boar spermatozoa supplemented with caffeine improved fertility [100]. Later, the same authors tested a combination of caffeine (1.15 mM) with the antioxidant compound β-mercaptoethanol (50 µM) but pregnancy rate was not modified (20 vs. 21% control and treatment respectively) after AI. However, litter size (10.0 ±1.0) almost doubled the data from control samples (5.7 ± 1.5) (*p* < 0.07) [78].

## 8. Antioxidants as a Therapy to Improve Reproduction Outcome

Sperm produce ROS as consequence of high aerobic metabolism. ROS production at nonphysiological levels overwhelm cellular scavenger systems and result in deleterious effects, such as lipid and protein peroxidation and DNA damage. Infertile men are known to possess pathological ROS levels, leading to sperm DNA fragmentation and lower ART outcome [29]. Thus, to deal with ROS overproduction and their deleterious effects at cellular levels in the male reproductive system, different strategies have been tested: (i) antioxidant oral consumption and (ii) antioxidants used as additives to media during ART.

Literature concerning the use of compounds with antioxidant activity and the improvement of sperm function is extensive. Nevertheless, others have found negative results [101,102], questioning the beneficial impact of antioxidant prescription and arguing that there is not clear evidence supporting prescription of antioxidants [103] or even that the over exposure to antioxidants can lead to other pathologies [104]. Others have found that administration of high doses of antioxidants have harmful effects on health [105,106]. Most trials have the handicap of using a lower number of men or are not double-blind or placebo-controlled. Moreover, the heterogeneity of the treatments and concentrations used as well as the experimental design make it hard to establish solid conclusions. Studies with greater numbers of patients should be performed, including large control groups to address the effects of oral antioxidant consumption on reproductive outcome. Moreover, arbitrary formulations of antioxidants should be avoided and classical pharmacological concentration-dependent experiments should be performed in order to find effective concentrations of antioxidants. Rather than by oral consumption, better reproductive outcome results are described when antioxidants were implemented in ART, especially during cryopreservation-thawing procedures. Antioxidant supplementation decreased LPO and improved reproductive outcome. Antioxidant concentration should be adapted to each form of ART. The future of antioxidant therapy to improve ART involves the development of nonintrusive technologies that can discern between sperm with or without lipid peroxidation or DNA damage, allowing physicians to inject healthy sperm into the oocyte by ICSI.

## Figures and Tables

**Table 1:** Effects of oral antioxidant intake on infertile men’s reproductive outcome.

Antioxidant Type and Daily Dose	Period Intervention (months)	ART	Relevant Findings	Participants	Problem	Reference
Astaxantin (16 mg)	3	NI and IUI	↑ Pregnancy rate 54.5% (5/11) vs. 10.5% (2/19) placebo group	30	Infertile	[39]
LC (1 g twice) LAC (0.5 g twice)	3		↓ ROS levels ↑ Pregnancy (11.7%) in patients with abacterial-PVE with normal values of leucocytes It didn´t improve pregnancy (0%) in abacterial-PVE patients with high levels of leucocytes	54	PVE	[40]
Nonsteroidal anti-inflammatory + carnitine (Carnitene, 2 g + Nicetile 1 g) Carnitine (Carnitene, 2 g + Nicetile 1 g) Nonsteroidal anti-inflammatory Nonsteroidal anti-inflammatory + carnitine (Carnitene, 2 g + Nicetile 1 g)	2 + 2 4 4 4		23.1% pregnancy 0% pregnancy 6.2% pregnancy 3.8% pregnancy	98	PVE with ↑ levels of leucocytes	[41]
LC (3 g), LAC (3 g), LC (2 g) + LAC (1 g)	6	NI	↑ Total oxyradicals scavenging capacity of seminal fluid ↑ Sperm motility and concentration. Pregnancy rate was not modified	60	Asthenozoospermic	[42]
LC (1 mg), fumarate (725 mg), LAC (500 mg), Fructose (1000 mg), CoQ10 (20 mg), Vitamin C (90 mg), Zinc (10 mg), Folic acid (200 μg), Vitamin B12 (1.5 μg)	6	NI	↑ Achieved pregnancy in treated men 22.2% (10/45) vs. 4.1% (2/49) non treated group	104	Oligo-and/or astheno-and/or teratozoospermia	[43]
LC fumarate (2 g), LAC (1 g) Clomiphene citrate (50 mg) and a complex of vitamins and microelements	3–4	NI	↑ Sperm concentration No modification in pregnancy rates	173	Oligo- and/or asteno- and/or teratozoospermia	[44]
LC fumarate (1 g), Acetyl-L- carnitine HCl (0.5 g) Fructose (1 g), Citric acid (50 mg), Vitamin C (90 mg), Zinc (10 mg), Folic acid (200 µg), Selenium (50 µg), Coenzyme Q-10 (20 mg) Vitamin B12 (1.5 µg)	6	NI	↑ Sperm concentration,% of sperm motile or progressive motility as well as sperm with normal morphology Treated men achieved 29% pregnancy versus 17.9% in the placebo group	90	After performed a varicocelectomy	[45]
Vitamin E (600 mg)	3	IVF	Improvement of zona pellucida binding test No effect on ROS levels No alteration on seminal plasma vitamin E levels	30	Infertile	[46]
Vitamin E (300 mg)	3	NI	21% of men had improved sperm motility and achieved pregnancy where 81.8% of pregnancies finished with a live birth	52	Asthenospermic	[47]
Vitamin E (200 mg)	1	IVF	↓ Sperm LPO ↑ Fertility rate: 19.3 ± 23.3 pre-treatment versus 29.1 ± 22.2 post-treatment	15	Normospermic infertile	[48]
Vitamin E (1 g) Vitmin D (1 g)	2	ICSI	76.3% respond to the treatment with ↓DNA damage ↑ Pregnancy rate (6.9 vs. 49.3%) ↑ Implantation rate (2.2 vs. 19.2%) Equal embryo quality	38	Infertile men non responding to ICSI	[49]
Vitamin E (400 IU) Selenium (200 µg)	3.5	NI	10.8% pregnancy	690	Infertile	[50]
Vitamin E (400 IU), Vitamin C (100 mg), Lycopene (6 mg), Zinc (25 mg), Selenium (26 μg), Folate (0.5 mg), Garlic (1000 mg)	3	IVF-ICSI	Doubled pregnancy rate (63.9 vs. 37.5%), Doubled implantation rate (46.2 vs. 24%) Doubled viable pregnancy rate (38.5 vs. 16%)	60	Infertile men with ↑ levels of DNA fragmentation and poor motility and membrane integrity	[51]
Zinc sulphate (220 mg)	4	NI	21.4% (3/14) of patients achieved pregnancy Zinc levels were increased in seminal plasma	14	Human	[52]
Zinc sulphate (500 mg)	3	NI	Improved pregnancy (22.5%) vs. placebo (4.3%) Zinc levels were not modified on seminal plasma	100	Asthenozoospermic	[53]

NI: natural insemination, IVF: in vitro fertilization, ICSI: intracytoplasmic sperm injection, IU: international unit, PVE: prostate-vesiculo-epididymitis, LC: L-carnitine, LAC: L-acetyl-carnitine, LPO: lipid peroxidation, ↑ increase, ↓ decrease.

**Table 2:** Antioxidants used as additives in different ART and their reproduction outcomes.

Antioxidant Type and Dose	Administration	Procedure	Principal Results Found	Stress	Specie	Reference
BHT 0.4 mM	In vitro	IVF	↑ Sperm survival ↓ Sperm MDA levels at the concentration ↑ Embryo develop 28.8% treated vs. 15.8% control	Cryopreservation	Boar	[75]
BHT 1 mM BHT	In vitro	IUI	↑ Pregnancy rate (86.7 vs. 63.6%), ↑ nº of gilts farrowing (86.7 vs. 45.4%) ↑ nº of piglets born (10.8 ± 1.6 vs. 8.2 ± 2.2)	Cryopreservation	Boar	[76]
BHT (2 mM), Ascorbic acid (8.5 mg/mL), Cysteine (5 mM), Hypotaurine (10 mM)	In vitro	AI	↓ Sperm LPO ↑Fertility: ascorbic acid (42.85%), BHT (35.71%), control (26.38%)	Cryopreservation	Goat	[77]
Caffeine (1.15 mM), β-mercaptoethanol (50 µM)	In vitro	AI	No effect on pregnancy rate ↑ Litter size in treated samples (10.0 ±1.0) vs. control (5.7 ± 1.5)	Cryopreservation	Boar	[78]
CAT (200 IU/mL)	In vitro		No differences on sperm parameters ↓ 2 pronucleus zygote (25.5% control vs. 13.2% treated)↓ Cleaved embryos: 7.6% treated vs. 16.7% control	Cryopreservation	Ram	[79]
Carnitine, Folic acid, Lycopene, Selenium, Vitamin C, Vitamin E, Zinc	Oral	NI	Duplicate fertilization rate (73.7 vs. 35.2%) Halved fetus reabsorption (9 vs. 18%)	Gpx5 knockout (KO) + Scrotal heat stress (KO + HS)	Mouse	[58]
Cysteine (2 mM)	In vitro	IUI	↑ SOD and CAT levels and = MDA levels ↑ Sperm total motility ↓ acrosome abnormalities Slight tendency to improve (*p* ˃ 0.05) non-return rate 74.54 (41/55) in comparison to control 57.14 (28/49)	Cryopreservation	Bull	[80]
Cysteine (10 mM), Rosemary extract (Rosmarinus officinalis). or a combination of both	In vitro	IVF	↑% sperm motility and progressive motility ↓ Acrosome membrane damaged Rosemary yielded better cleave% without affects blastocysts	Cryopreservation	Boar	[81]
Cysteine (5 mM) Trehalose (25 mM)	In vitro	IUI	No improvement of antioxidants features No differences on non-return rate was found after IUI	Cryopreservation	Bull	[82]
Cysteamine (5 µM), Lycopene (500 µg/mL)	In vitro	IUI	No differences on non-returned rate	Cryopreservation	Bull	[83]
EGCG (50 mg/kg)	Intraperitoneal		Restore testicular function ↓ LPO and protein carbonyl levels ↑ Number of pups by littler	Ionizing radiation	Rat	[66]
GSH (0.5 and 1.0 mM) GSH 0.5 mM + SOD 100 U/mL	In vitro	IUI	Equal nonreturn rates	Cryopreservation	Bull	[84]
Melatonin (1 mM)	In vitro	IVF	↑ Sperm viability rates ↑% of total motile and progressive motile spermatozoa ↑ DNA integrity Faster first embryonic division	Cryopreservation	Ram	[85]
Metformin (50 to 5000 µM)	In vitro	IVF	Duplicate fertilization rate and embryo development	Cryopreservation	Mouse	[86]
NAC (1–10 mM)	In vitro	ICSI	Decrease ROS ICSI outcome wasn’t modified	Thawing + H_2_O_2_	Bull	[87]
NAC (10 μM), LAC (10 μM), α-Lipoic Acid (5 μM)	In vitro	IVF	↓ Embryo intracellular levels of H_2_O_2_ Accelerated embryo development and blastocysts ↑ TE and ICM cell numbers	Incubation under 20% O_2_	Mouse	[88]
Taurine (2 mM)	In vitro	IUI	↓ GSH and SOD levels but ↑ five-fold CAT levels ↑ MDA levels = nonreturn rates	Cryopreservation	Bull	[80]
Zinc chloride (10 µg/mL), d-aspartic acid (500 µg/mL) Coenzyme Q10 (40 µg/mL)	In vitro	IVF	↑% of total spermatozoa motile and progressive motility ↓ Sperm and blastomeres DNA fragmentation ↑ 8-cells blastocyst: 51.4% treatment vs. 37.1% control	Cryopreservation	Bull	[89]

IVF: in vitro fertilization; AI: Artificial Insemination; IU: international unit; NI: natural insemination; IUI: intrauterine insemination; TE: trophectoderm; ICM: inner cell mass; BHT: butylated hydroxytoluene; CAT: catalase; GSH: reduced glutathione; NAC: N-acetyl-l-cysteine; LAC: l-acetyl-carnitine; EGCG: epigallocatechin-3-gallate, ↑ increase, ↓ decrease.
